# Study on Network Structure and Heat Resistance in Air of Boron-Modified Phenolic Resin Aerogel

**DOI:** 10.3390/polym17070860

**Published:** 2025-03-24

**Authors:** Tengfei Wu, Degang Wang, Qin Wang, Xiaolong Chen, Jie Ding, Xizhuo Yan

**Affiliations:** 1 School of Materials Science and Engineering, Wuhan University of Technology, Wuhan 430070, China; wutengfei@whut.edu.cn (T.W.); c15970166306@163.com (X.C.); rheinlanna@whut.edu.cn (X.Y.); 2 Shenyang Aircraft Design Research Institute, Shenyang 110000, China; darwin_wang@163.com (D.W.); wq35050212@126.com (Q.W.)

**Keywords:** boron modification, heat resistance in air, network structure, phenolic resin aerogel

## Abstract

Phenolic aerogel is one of the most widely used lightweight thermal protective materials at present. With changes in the application environments, higher requirements are put forward for the heat resistance and mechanical properties of phenolic aerogel. In this paper, boric acid was used to modify phenolic resin, and then boron-modified phenolic aerogel was prepared. The chemical structure of modified phenolic resin was studied by infrared spectroscopy (FTIR). The microstructure, thermal stability, heat resistance in air, and compression resistance of phenolic aerogel were studied by volume shrinkage, scanning electron microscope, thermogravimetric analysis, high-temperature combustion test, and mechanical test. The results showed that the modification introduced boron oxygen bonds on the phenolic main chain. The compatibility difference between boron and phenolic resin with different content has a significant impact on the performance of phenolic aerogel. When boron content is 5–10% of phenolic resin, the network structure and thermal stability of phenolic aerogel can be significantly improved, and the maximum compressive strength of phenolic aerogel can also be improved. Boron-modified phenolic aerogel is expected to play an important role in the field of thermal insulation.

## 1. Introduction

Phenolic aerogel possesses a low density, good thermal insulation, low cost, and high specific surface area [1,2], and has a wide range of applications in the field of thermal protection [3,4,5]. With the changes in the service environment and operating conditions of aircraft, new thermal and mechanical requirements have been imposed on phenolic aerogel, a traditional thermal protection material [6,7]. Phenolic aerogel itself suffers from inadequate mechanical properties and insufficient oxidation resistance [8,9], which renders it ill-suited to more challenging use environments.

In recent years, a significant number of researchers have enhanced the oxidation and ablation resistance of phenolic aerogels through various modifications [10,11,12,13]. For instance, the addition of inorganic particles to phenolic aerogels has been shown to enhance their thermal stability and oxidation resistance. Li et al. [14] enhanced the thermal stability properties of phenolic aerogels by adding TiB-B4C particles. Other researchers have added nanoclay by means of organic modification and investigated the effect of nanoclay addition on mechanical, thermal stability, and ablative properties [15]. However, the addition of inorganic particles increases the bulk density and heat transfer capacity of phenolic aerogels, and the uniformity of dispersion is difficult to ensure [16].

Besides the introduction of nanoparticles, a part of the researchers have designed phenolic aerogels at the molecular level by means of chemical modification [17] in order to improve the various properties of phenolic aerogels and also to avoid the phase separation phenomenon. At present, a greater number of researchers have modified the properties of phenolic aerogels by organosilicon [18,19]. Huang et al. [20,21] enhanced the flexibility of phenolic aerogels by branching organosilicon backbones onto phenolic molecular chains. This development addressed the issue of brittleness in phenolic aerogels, whilst concurrently conferring hydrophobicity and compressibility. Fu et al. [22] enhanced the thermal stability and ablation resistance of phenolic aerogels by introducing silica–zirconium elements. Yang et al. [23] prepared phenolic aerogels with good stability and thermal insulation properties and good mechanical properties by polyurethane modification of phenolic aerogels. Gao [24] made a significant improvement in the thermal stability of phenolic aerogels by inserting B-O bonds with higher bond energies into phenolic aerogels.

The purpose of this article is to modify phenolic resin without increasing its density, in order to improve its crosslinking structure and high-temperature oxidation resistance. The compatibility issue between boron with different contents and phenolic resin was studied. The effects of boron content on the microstructure, physical properties, high-temperature oxidation resistance, and mechanical properties of phenolic aerogels were studied. The mechanism of boron improving the thermal stability of phenolic aerogels was also discussed.

## 2. Experimentation

### 2.1. Materials

Phenolic resin solution (PR, 35% ethanol as solvent) was purchased from Henan Hengyuan New Materials Co., Ltd. in Zhengzhou, China. Hexamethylenetetramine (HMTA), purchased from Henan Borun Casting Materials Co., Ltd. in Zhengzhou, China. Boric acid (H_3_BO_3_) and anhydrous ethanol (analytically pure) were purchased from Sinopharm Chemical Reagent Co., Shanghai, China. All reagents require no further processing.

### 2.2. Preparation of Boron-Modified Phenolic Aerogel

**Figure 1:**
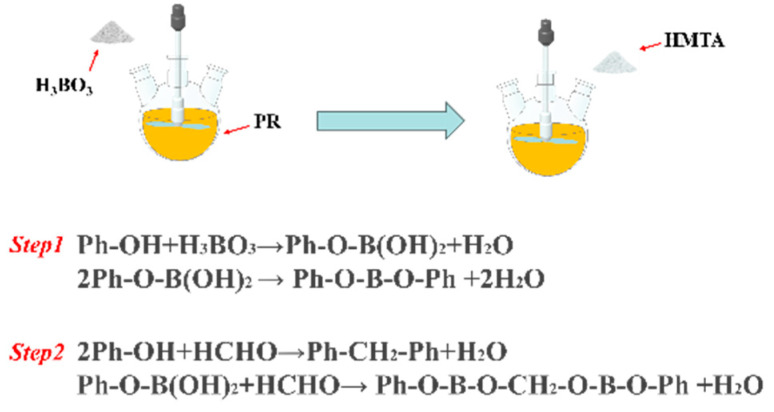
The synthesis mechanism of BRx.

The phenolic resin solution was mixed with boric acid in a three-necked flask and stirred in a water bath at 30 °C for 1 h. The modified phenolic solution was mixed with HMTA at a mass ratio of 7:1 and continued to be stirred for 1 h. The boron-modified linear phenolic resin solution was obtained. The preparation process is shown in Figure 1. The main reaction process is as follows:

(1) Boric acid (H_3_BO_3_) reacts with the phenolic hydroxyl group (Ph-OH) of linear phenolic resin to form boronic ester bonds. Further dehydration forms a boronic ester crosslinked structure.

(2) The added HMTA decomposes into formaldehyde (HCHO), which condenses with unreacted phenolic hydroxyl groups and boronic acid esters in the resin to form a boron-containing crosslinked network.

**Figure 2:**
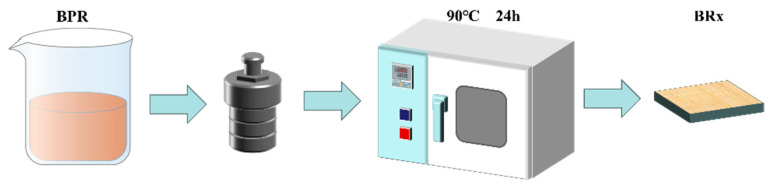
Boron-modified phenolic aerogel preparation.

The prepared boron-modified linear phenolic resin solution was placed in a reactor. Placed in a 90 °C oven sol–gel reaction 24 h, under the combined action of high temperature and ring tension, the C-N bond in HMTA breaks, and the C atom in phenolic aldehyde is bonded and crosslinked to form a wet gel. After drying at 50 °C and atmospheric pressure for 12 h, the material known as boron-modified phenolic aerogel was successfully obtained. The flowchart is shown in Figure 2. The various phenolic resins are denoted by the code BRx (x = 5%, 10%, 15%, 20%). The different synthetic formulations are shown in Table 1.

### 2.3. Characterisation

In order to demonstrate the successful crosslinking of boron-modified phenolic aerogels, the molecular structures of PR and BRx were analyzed by observing the absorption spectra of PR and BRx using Fourier Transform Infrared Spectroscopy (FTIR) in the range of 500–4000 cm^−1^.

The bulk densities of PR and BR_x_ were calculated from measurements based on sample volume and weight.

(1) $$\rho =\frac{m}{v}$$

where *m* is the mass of the dried sample and *v* is the volume of the dried sample.

(2) $$S=\frac{V_{1}-V_{2}}{V_{1}}\;*\;100\%$$

where *S* is the volume shrinkage rate of the sample after drying, V_1_ is the volume of the mold cavity, and V_2_ is the volume of the sample after solidification and drying.

In order to investigate the effect of different contents of boron on the crosslinking structure of phenolic aerogels, the microstructure of the samples was analyzed using scanning electron microscopy (SEM) to observe the crosslinking structure and microporous distribution of the samples.

Thermogravimetric analysis on PR and BRx was performed using a thermogravimetric analyzer in a nitrogen environment with a heating rate of 10 °C/min and a maximum temperature of 1000 °C

The samples of different components were prepared into cubic blocks of regular shape, their mass was measured, and their residual mass was measured after static pyrolysis at 1000 °C for 20 min in a muffle furnace to study the high-temperature antioxidant properties of phenolic aerogels.

(3) $$M=\frac{m_{1}-m_{2}}{m_{1}}\;*\;100\%$$

where *M* is the mass residual rate, *m*_1_ is the mass before heat treatment, and *m*_2_ is the mass after heat treatment.

The compressive properties of phenolic aerogels were measured with a3300 series dual-column desktop electronic universal machine tester at a loading rate of 2 mm/min. The size of the test sample is 20 mm × 10 mm × 10 mm.

## 3. Results and Discussion

### 3.1. Synthesis of Boron-Modified Phenolic Resin

**Figure 3:**
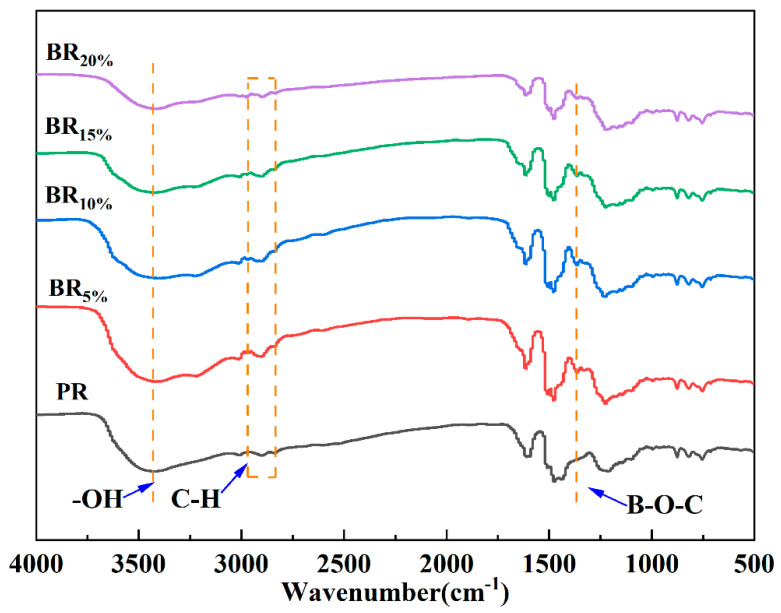
FTIR spectra of different component samples.

The infrared spectra of PR and BR_x_ are shown in Figure 3. The results showed that compared with the infrared spectrum of PR, BR_x_ exhibited a significant absorption peak near 1359 cm^−1^, which was attributed to the B-O-C stretching vibration, consistent with the literature [24]. The other absorption peaks of BR_x_ are similar to those of PR, and the distribution of characteristic peaks is as follows. The characteristic peaks of O-H and -CH_2_OH of phenolic groups are around 3500 cm^−1^ (broad peak). The absorption peak range of C-H stretching vibration in -CH_2_- is 2800 cm^−1^ to 2950 cm^−1^. The stretching vibration peaks of the C=C double bond in the benzene ring appear at 1610 cm^−1^ and 1510 cm^−1^ [25]. The absorption peaks of aliphatic C-O stretching vibration are at 1130 cm^−1^ and 1005 cm^−1^, respectively. The ortho substitution peak and para substitution peak of the benzene ring are located at 760 cm^−1^ and 870 cm^−1^, respectively. The C-N stretching vibration peak at 1256 cm^−1^ indicates that the curing mechanisms of PR and BPR are the same, both of which are cured through the thermal decomposition of curing agent HMTA to produce dimethyl hydroxylamine, formaldehyde, and PR crosslinking. It can be confirmed that boric acid has been incorporated into the structure of phenolic resin.

### 3.2. Formability of Boron-Modified Phenolic Aerogel

**Figure 4:**
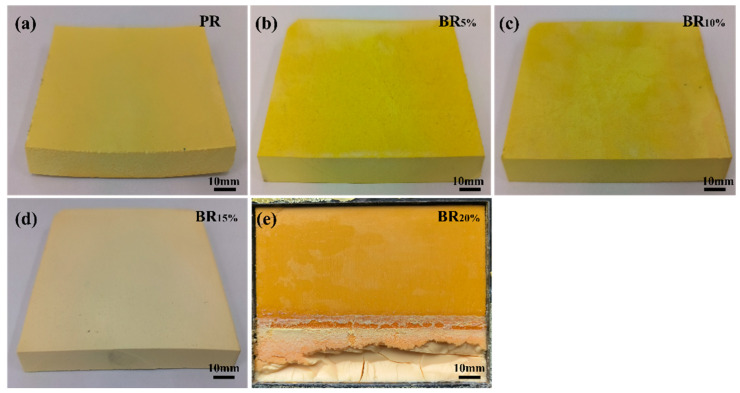
Macroscopic topography.

Figure 4 shows the macro morphology of phenolic aerogel. The physical property diagram of phenolic aerogel is shown in Figure 4a–e, and the physical property parameters of different samples are listed in Table 2. From Figure 4a, it can be seen that PR exhibits significant volume shrinkage and warping deformation during the curing process, indicating the presence of significant internal stress during the curing process, leading to the deformation of its final form. Figure 4b–d shows the effect of different boron content on the formability of phenolic aerogels. When the boron content is between 5% and 15% of the phenolic resin, the phenolic aerogel shows good formability, and there is no significant shape change or deformation after curing, indicating that when the boron content is moderate, the boron and the phenolic resin are well crosslinked, which can effectively enhance the formability of the aerogel. However, Figure 4e shows that when the boron content reaches 20%, the phase separation phenomenon between phenolic resin and boric acid becomes obvious, resulting in the appearance of two structures in the cured sample, and the overall structural stability decreases, affecting the molding effect. The results show that the change in boron content has a significant effect on the formability of phenolic aerogels. When the boron content is low, the phenolic aerogel can maintain good formability and stable shape after curing. However, when the boron content is too high, phase separation will occur between the resin and boron, which will significantly reduce the uniformity and molding effect of the aerogel. In general, proper boron addition can improve the molding performance of phenolic aerogels, but excessive boron content will have a negative impact on the overall performance of the gel.

Table 2 shows that the density of PR is 0.537 g/cm ^3^, and its volume shrinkage rate is 34.723%. After adding boron, the shrinkage rate of PR significantly decreased. When the boron content is 5–15%, the shrinkage of BR_x_ is low, especially the shrinkage of BR_10%_ is only 18.35%, which is about 16% less than PR, indicating that the addition of boron improves the shrinkage behavior of phenolic aerogels and significantly improves their dimensional stability. The results showed that the appropriate amount of boric acid could effectively reduce the volume shrinkage during the curing process, making the aerogel maintain better morphology and structural stability. When the boron content is low, the density of BR_x_ is lower than that of PR, indicating that the introduction of boron may lead to a change in micropore structure in the aerogel during the curing process. This change may increase the porosity inside the aerogel, resulting in a decrease in the overall density. These data show that the addition of boron not only affects the dimensional stability of the aerogel, but also may affect its microstructure, thus affecting its macro physical properties. When the boron content reaches 20%, obvious phase separation occurs, which leads to uneven formation of aerogels, and the density and shrinkage cannot be measured accurately. The phase separation phenomenon shows that too high boron content will lead to a decrease in the compatibility between phenolic resin and boric acid, thus affecting the stability and uniformity of the aerogel, leading to a significant decline in its physical properties. Therefore, the additional amount of boric acid needs to be strictly controlled to ensure the good overall performance of phenolic aerogel.

### 3.3. Microstructure of Boron-Modified Phenolic Aerogel

**Figure 5:**
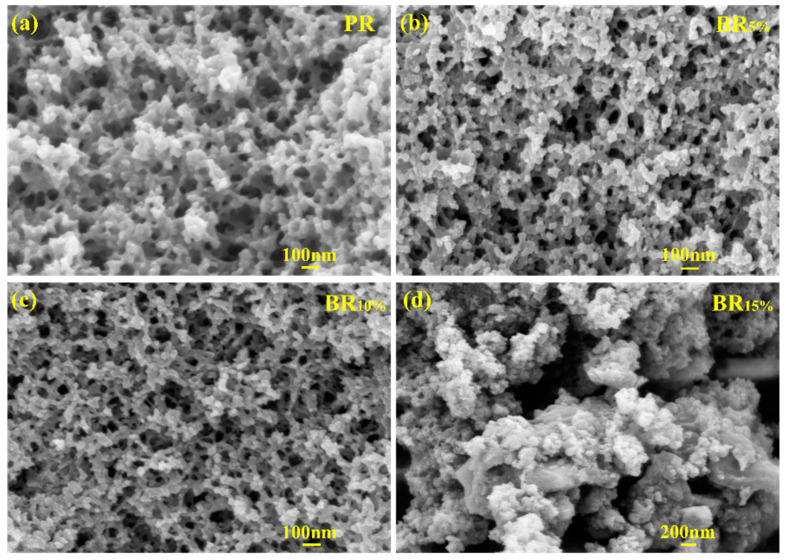
SEM images of PR and BR_x_ (**a**) PR, (**b**) BR_5%_, (**c**) BR_10%_, (**d**) BR_15%_.

The scanning electron microscopes of PR and BR_x_ are shown in Figure 5a–d. Figure 5a shows the microstructure of PR, the PR aerogel shows a typical three-dimensional network porous structure, and the morphology of PR particles is uniform, showing a good pore distribution. Figure 5b,c shows that the microstructure of phenolic aerogel changes with the change in boron content. When the boron content is in the range of 5–10%, the pore size of the aerogel decreases gradually, and the pores are evenly distributed. The structure shows that the introduction of boron in this range helps to improve the crosslinking of phenolic resin, making the microstructure of the aerogel more stable, and the pore size smaller and more uniform. When the boron content reaches 15%, the pore size of the aerogel increases significantly, the framework becomes larger and irregular, and the uniformity of pore size distribution decreases significantly. The occurrence of this phenomenon can be attributed to the microphase separation between boric acid and phenolic resin during the curing process. At higher boron concentrations, the compatibility between boron and phenolic resin begins to decrease, leading to incomplete crosslinking reactions and the formation of larger pores and rough skeletal structures. The results show that the introduction of boron has a significant effect on the microstructure of phenolic aerogels. When the boron content is introduced in a lower range, the interaction between boron and resin promotes a tighter crosslinked network structure, resulting in a decrease in pore size and uniform distribution. When the boron content is high, its compatibility with phenol formaldehyde will decrease, resulting in a larger crosslinked skeleton and uneven distribution of pore size [26].

### 3.4. Thermal Stability of Boron-Modified Phenolic Aerogel in N_2_

**Figure 6:**
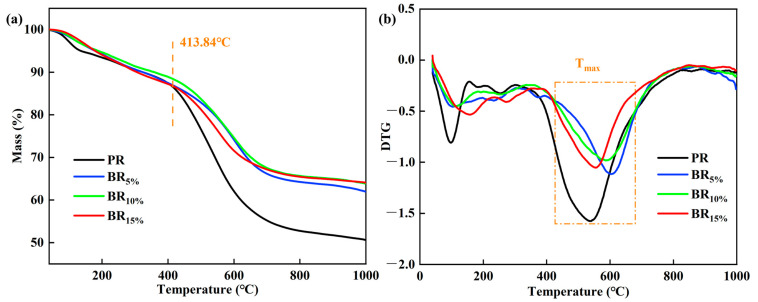
TG curve (**a**) and DTG curve (**b**) of PR and BRx aerogels.

The TG-DTG curves of phenolic aerogel are shown in Figure 6a and Figure 6b respectively. From the TG curve in Figure 6a, it can be seen that phenolic aerogels and hybrid phenolic aerogels have similar thermal decomposition behavior. From the DTG curve, it can be seen that the thermal decomposition rate of phenolic aerogel has two main peaks. At 0–200 °C, due to the decomposition of free phenol and the volatilization of residual solvent ethanol in the phenolic aerogel, there is a high decomposition rate. However, compared with phenolic aerogels, the decomposition rate of hybrid aerogels decreased. At this stage, the volatile mass of all aerogels is less than 20%. In the stage of 200–400 °C, mainly ph-OH dehydration and C-N fracture, all phenolic aerogels show relatively stable thermal decomposition behavior, and the decomposition rate is relatively slow. A wide peak appears in the range of 400–700 °C. This stage is mainly due to the fracture of the methylene bond, accompanied by the collapse of the phenolic aerogel skeleton, which leads to a relatively obvious increase in the decomposition rate and a rapid decline in weight. After 700 °C, the phenolic aerogel reached a relatively stable state, and the mass loss rate was less than 5%.

From TG and DTG curves, it can be seen that the mass loss of PR is close to 50%, while the mass loss rate of all hybrid phenolic aerogels is within 40%, and the residual rate is increased by about 10%. PR reached the maximum decomposition rate at about 530 °C, while the maximum decomposition rate of hybrid gas gel increased to about 600 °C. The thermal stability of materials is usually expressed by the temperature T_5%_ of weight loss 5% and the maximum weight loss rate T_d, max_. The T_5%_ and T_d, max_ of hybrid phenolic aerogels are significantly higher than that of phenolic aerogels, showing better thermal stability. The main reason is that the bond energy of B-O in a hybrid gel is much higher than that of C-O. Therefore, the chemical bond breaking of hybrid aerogel requires a higher temperature, so it attains better thermal stability.

### 3.5. Heat Resistance in Air of Boron-Modified Phenolic Aerogel

**Figure 7:**
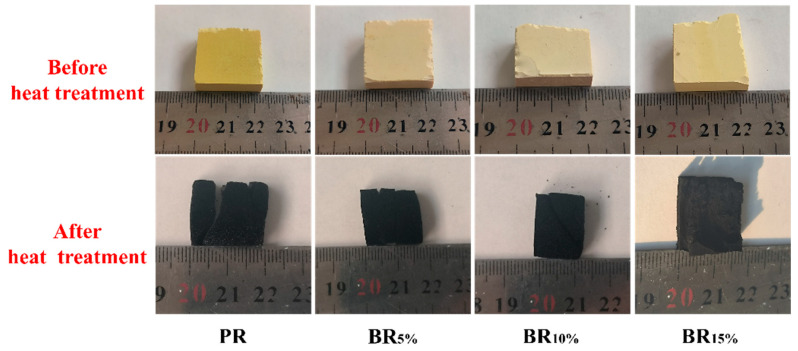
Pictures of PR and BR_x_ aerogels before and after pyrolysis.

Photos of aerogel samples before and after heat treatment are shown in Figure 7. Table 3 shows the mass residual rate of the samples after heat treatment. Figure 7 shows the changes in phenolic aerogels with different boron contents after high-temperature heat treatment. From these images, it can be clearly seen that the PR samples showed significant volume reduction after heat treatment, and severe cracks appeared on the surface, with residues appearing on the surface of the samples. This phenomenon indicates that phenolic aerogel may have experienced severe gas escape and volume contraction during heat treatment. The BR_5%_ and BR_10%_ samples showed relatively good performance after heat treatment, with intact surfaces and fewer cracks. It can be seen from the photos that the surfaces of BR_5%_ and BR_10%_ aerogels are relatively dense. This indicates that although there was slight volume shrinkage and gas escape during the heat treatment process of these two samples, the overall structural stability was high, and no serious cracks or damage occurred. Compared with PR samples, the thermal stability of these two samples in air is significantly improved, indicating that moderate boron content is helpful in improving the thermal stability of aerogels. In contrast, the BR_20%_ sample showed significant surface collapse after heat treatment, with larger areas of depression and more cracks than the BR_5%_ and BR_10%_ samples. Boron in phenolic aerogel reacts with oxygen in the air at high temperatures to produce B_2_O_3_. The formation of B_2_O_3_ can protect the surface of aerogel to a certain extent and slow down the rate of thermal decomposition. However, when the boron content reaches 15%, the compatibility between boron and phenolic aerogel begins to decline, leading to the decline in structural uniformity of phenolic aerogel, which cannot ensure that the surface of phenolic aerogel is evenly covered with B_2_O_3_ [27]. Therefore, the surface structure of BR_20%_ is relatively loose, and its thermal stability is significantly reduced. The results showed that the excessive boron content not only affected the microstructure of the aerogel, but also led to the decline in the thermal stability of the phenolic aerogel in air.

The data in Table 3 show that the mass residual rate of PR samples after heat treatment is about 36.5%, indicating that there is a significant loss of mass in PR after heat treatment. However, with the increase in boron content, the mass residual rate of BR_x_ samples increased significantly, indicating that the introduction of boron had a significant impact on the thermal stability of phenolic aerogels. Among them, the mass residual rate of BR_5%_ is 49.72%, and BR_10%_ is 54.92%. The mass residual rate of BR_10%_ is about 20% higher than that of the PR sample. This result shows that the introduction of boron effectively slows down the decomposition rate of aerogel during heat treatment, and significantly enhances its thermal stability. However, the mass residual rate of the BR_15%_ sample decreased, indicating that too high boron content may adversely affect the thermal stability of the aerogel. This phenomenon may be caused by the non-uniformity of boron oxide generated in the sample. The introduction of boron oxide (B_2_O_3_) has been proven to be able to effectively improve the thermal stability of phenolic aerogels [28]. B_2_O_3_ can form a protective film on the surface of aerogel to slow down the escape of gas and collapse of the aerogel structure during pyrolysis. In this way, boron oxide effectively inhibits the pyrolysis of aerogels at high temperatures, thus improving the thermal stability of aerogels. The change in boron content also has a significant effect on the decomposition behavior of phenolic aerogels. An appropriate amount of boron can be evenly dispersed in the structure of phenolic aerogel to enhance its overall structural stability. However, when the boron content is too high, the dispersion of boron will decline, forming an uneven structure, which will affect the decomposition behavior of phenolic aerogels in the thermal environment. The results showed that the addition and distribution of boron affected the thermal stability of phenolic aerogels. In the process of heat treatment, uniformly dispersed boron can improve the thermal stability of phenolic aerogel in air, while excessive boron can cause a decrease in the mass residual rate of phenolic aerogel after heat treatment.

### 3.6. Mechanical Properties of Phenolic Aerogel

**Figure 8:**
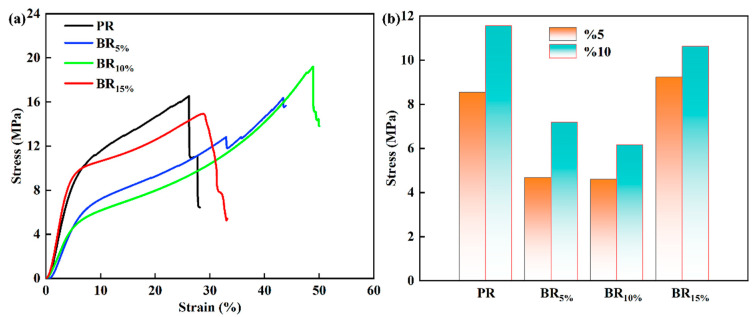
(**a**) Stress–strain curve of PR and BR_x_. (**b**) Compressive strength of PR and BR_x_ under different deformations.

According to Figure 8a, the stress–strain curves of PR and BRx exhibit typical nonlinear concave compression responses. When the deformation is low (<6%), the stress–strain curves of PR and BRx show a linear increase, indicating elastic deformation behavior; As the loading stress increases, the deformation of the material shows significant differences, with the PR deformation collapsing at around 25% and the stress rapidly decreasing. The compressive strength of BR10% is significantly improved, and the deformation under maximum load is increased. While the mechanical strength of the surface material increases, the toughness is also improved. The deformation of BR15% is relatively high, but the compressive strength is low, exhibiting poor mechanical properties. Figure 8b shows the mechanical properties of the material under 5% and 10% deformation. The strength of PR is relatively high, while BRx exhibits lower mechanical strength at low deformation, indicating that PR exhibits higher strength and a certain degree of rigidity at low deformation. The introduction of B changed the crosslinking structure inside the phenolic aerogel and increased its toughness. As the B content further increases, microphase separation and particle accumulation phenomena occur, resulting in lower strength and deformation of BR15%. Based on the microstructure, it can be concluded that at this point, there is a severe accumulation of particles inside the material, and the distribution of crosslinking networks is uneven, which seriously affects the compressive strength of the material and significantly reduces the mechanical properties of BRx.

## 4. Conclusions

In this paper, boron-modified phenolic aerogels were prepared, and the compatibility between boron and phenolic resin and the effect of boron content on the properties of phenolic aerogels were studied. It was found that the physical properties of the modified phenolic aerogel could be changed by adjusting the boron content, the bulk density was 0.507 to 0.642 g/cm^3^, and the volume shrinkage was reduced from 34.72% to 18.35%. When the boron content is 5–10% of the phenolic resin, the phenolic aerogel shows a more uniform microporous structure. Boric acid crosslinks with phenolic resin to form B-O, whose bond energy is significantly higher than C-O, and produces B_2_O_3_ under high-temperature environments, which has a certain protective effect on the interior of the aerogel, thus significantly improving the thermal stability and high-temperature oxidation resistance of boron-modified phenolic aerogel. After heat treatment at 1000 °C in a nitrogen environment, the carbon residue rate increases by about 10%. The residual quality rate in the air environment increased by about 20%. When boron content was 10%, the compressive strength of phenolic aerogel reached 19.20 MPa, which increased by about 16%. Appropriate boron content can improve the compressive strength and toughness of phenolic aerogels. In conclusion, the proper amount of boron-modified phenolic aerogels show good compatibility and good comprehensive properties, which are expected to be used as thermal insulation materials in aerospace applications.

## Figures and Tables

**Table 1:** Synthetic formulations of BR_x_.

Samples	Phenolic Resin (g)	H_3_BO_3_ (g)	B (wt%)
PR	100	0	0
BR_5%_	100	28.60	5
BR_10%_	100	57.20	10
BR_15%_	100	85.80	15
BR_20%_	100	114.40	20

**Table 2:** Physical property.

Samples	PR	BR_5%_	BR_10%_	BR_15%_	BR_20%_
Densities (g/cm^3^)	0.537	0.507	0.511	0.642	/
Volumetric shrinkage (%)	34.72	20.02	18.35	20.06	/

**Table 3:** Mass residuals after static pyrolysis of PR and BR_x_ aerogels.

Samples	PR	BR_5%_	BR_10%_	BR_15%_
Mass residual rate (%)	36.51	49.72	54.92	46.75

## Data Availability

Dates are contained in the main text.
